# Antidiabetic effects of *Scrophularia striata* ethanolic extract via suppression of *Pdx1* and *Ins1* expression in pancreatic tissues of diabetic rats

**DOI:** 10.1038/s41598-022-13698-w

**Published:** 2022-06-13

**Authors:** Armieti Babaiedarzi, Saba Ghanbari, Maryam Mehrad seresht, Modara Nasiri

**Affiliations:** 1 Tehran Medical Sciences, Islamic Azad University, Tehran, Iran; 2 Department of Biology, Faculty of Biological Sciences, North Tehran Branch, Islamic Azad University, Tehran, Iran; 3 Present Address: Research company located in Islamic Azad University Science And Technology Park, Araz Fidar Azma, Tehran, Iran

**Keywords:** Molecular biology, Physiology

## Abstract

One of the factors that causes severe metabolic imbalance and abnormal changes in many tissues, especially in the pancreas, is the pathological disease of diabetes mellitus. Therefore, in this study, the therapeutic effects of *Scrophularia striata* were investigated using an animal model in the control of diabetic injury and pancreatic complications caused by diabetes. A total of 66 rats (weight 220–250 g) were randomly divided into: Healthy Control group (rats without diabetes receiving Propylene glycol as solvent); Diabetic control group; 3 experimental healthy groups (receiving the extract with doses of 100, 200 and 400 mg/kg bw/day); 3 treatment groups; and3 pretreatment groups. Diabetes was induced in rats by intraperitoneal STZ (60 mg/kg bw). FBS, HbA1c and insulin were measured after 4 weeks. *Pdx1* and *Ins1* gene expression was assessed by RT-PCR. The histological evaluation was also performed with H&E staining. The data were analyzed by SPSS ver20 using ANOVA and Tukey tests. By treatment with *S. striata* ethanolic extract, these factors were close to the normal range. The expression of the *Pdx1* and *Ins1* genes increased in the treated rats with *S. striata* extract. Analysis of the obtained data indicates the effect of S. striata in improving the complications of diabetes in rats and can be considered for therapeutic purposes.

## Introduction

One of the oldest known disorders is Diabetes mellitus (DM) in humans^1^. A it is assumed that DM due to the inherent stresses of modern lifestyles and the increasing prevalence of diabetes is becoming a major public health problem affecting millions of people worldwide. According to the World Health Organization (WHO), by 2025, 300 million people will have diabetes. Diabetes mellitus is a pathological disease, and as a result causes severe metabolic imbalances and abnormal changes in many tissues, especially in the pancreas, where an important role in etiology is played by oxidative stress. High oxidative stress because of persistent and chronic high blood sugar is tested in diabetic and experimental animal models, thus destroying the activity of the antioxidant defense system and thereby producing free radicals^2–4^. In diabetes mellitus, changes in the defense mechanisms of endogenous free radical scavenging may lead to ineffective inhibition of oxygen-reactive species, leading to oxidative damage and tissue damage. Tissue damage has been suggested and streptozotocin acts as a diabetic agent due to its ability to destroy pancreatic β-cells, possibly by the mechanism of free radicals^5,6^.

The pancreas is the synthesis, storage and secretion site of insulin^7^. In the pancreatic islets, there is a complex signaling cascade for the secretion of glucose-stimulating insulin that includes ATP-sensitive potassium channels (K_ATP_). In the presence of glucose, an increase in the intracellular ATP/ADP ratio causes the K_ATP_ channels to close, resulting in the plasma membrane depolarization, extracellular calcium influx, and exocytosis activation. The cell plasma membrane islet has K_ATP_ channels, although most K_ATP_ channels are located on secretory granule membranes. The K_ATP_ channels of pancreas have four sulfonylurea-regulating receptor subunits (SUR1) and four potassium pore-formation subunits (K_ir_6.2)^8^. One of the chronic autoimmune diseases is type 1 diabetes (T1D) in which insulin-producing beta-cells in the pancreas are destroyed, resulting in chronic high blood sugar. Pancreatic exocrine abnormalities have been described in recent decades in terms of anatomy and function. It is not clear whether exogenous changes of in T1D are relevant to identical genetic, immunological, and environmental events that lead to the destruction of beta-cells and are secondary to functional β cell loss. Therefore, insulin acts as a trophic agent for the exocrine compartment^9^.

The main goal of caring for diabetic patients is to minimize the risk of microvascular and macrovascular complications by returning blood pressure, and lipid and glycemic profiles to normal. The particular aim for glycemic control is to reach glycated hemoglobin (HbA_1c_) to a normal range because good glycemic control is important to decrease the long-term microvascular complication risk in both type 1 and 2 diabetes^10^. HbA1c can be measured at any time of the day, regardless of the duration of the fast or the content of the previous meal. It can also be analyzed using a portable device with a small blood sample^11^. Glycated hemoglobin (HbA1c) analysis in blood enables evidence of a person’s average blood glucose level over the past 2–3 months, which is the red blood cell (RBC) half-life^12^.

There is ample evidence that β-cell regeneration and function in the adult pancreas are mediated partly by pancreatic and duodenal homeobox 1 (*Pdx1*), and that changes in expression are associated with the alterations in the expression of target genes, including insulin 1 (*Ins1*)^4^. Pdx1 is an important transcription factor needed for pancreatic development and maintains the distinctive function of β-cells, especially the regulation of normal glucose-insulin secretion^13,14^. The first transcription factor produced in the growing pancreas is *Pdx1*, and lack of this factor causes pancreatic agenesis because of the inability to generate a variety of duct, exocrine or endocrine cells. Conditional elimination of *Pdx1* from β cell formation by insulin Cre lines leads to hyperglycemia^15,16^. Previous studies have shown that *Pdx1* plays a major role in diabetes and that reducing the expression of *Pdx1* exacerbates diabetes^17,18^.

*Scrophularia striata*, known as “Teshnedari” from the Scrophulariaceae family, is one of the most important traditional herbal medicines that is widely used in western Iran^19^. This herb is an annual or perennial plant that has a zygomorphic flower and 5 petals flower, and the calyx has lobes and its fruit is a capsule with many seeds. It has been reported that *Scrophularia striata* has some medicinal effects including analgesic, antimicrobial, nephrotic, nitric oxide suppressant, antitumor, hepatic protection and anti-inflammatory effects^20–23^. Due to the medicinal effects of *Scrophularia striata* plants and their side effects, they have not been studied thus far. In this study, using an animal model, the effects of this plant were investigated in preventing diabetes-induced damage to the pancreas and its functions.

## Material and methods

### Ethics statement

All the animal maintenance and procedures were in accordance with recommendations established by the Animal Ethics Committee of the Islamic Azad University, as well as the United States NIH guidelines (publication no. 85-23). The Ethics Committee approved the protocols of The Islamic Azad University. All surgical procedures were carried out beneath deep anesthesia, and all efforts had been made to minimize suffering. The study is reported in accordance with ARRIVE guidelines (https://arriveguidelines.org).

### Animals

In this study, 66 Wistar rats (5 weeks aged) were purchased in the weight range of 180–200 g from Pasteur Institute, Tehran, Iran. They were transferred to the Laboratory Animal Breeding Center of Tehran Azad University Science and Technology Park. The animals were kept in a laboratory animal breeding center with fiberglass cages (15 × 30 × 35 cm) and a 12 h light/12 h dark cycle and temperature (22 ± 2 °C), light with a relative humidity range of 40–60%. The rats were fed particular food obtained from Behparvar Company, Tehran, Iran. Tap water was used to maintain hydration, and the experimental tubes were used in each cage as their water supply with sufficient water and food during the experiment. All research and laboratory animal care were performed by responding to the Guide for the Care and Use of Laboratory Animals (National Research Council, 2012). When their weight reached 220–250 g, the experiment was started.

### Selection of diabetic rats by inducing streptozotocin

Streptozotocin (STZ; Sigma Aldrich, USA) was used to induce diabetes. we used 0.1 M sodium citrate buffer (pH = 4.5) as STZ solvent. STZ was dissolved at a dose of 60 mg/kg body weight and injected intraperitoneal into rats during single STZ injection^24^. Blood glucose was monitored 48 h after STZ injection. To measure the inactive serum glucose level glucometer tapes (SD CODEFREE BLOOD GLUCOSE METER TEST STRIPS 50 T; SD Biosensor Inc, South Korea) were used on the rat tail vein. If the measured blood glucose was higher than 300 mg/dl, we considered diabetic rats^25^.

We used 0.1 M sodium citrate buffer (pH = 4.5) as STZ solvent. STZ was dissolved at a dose of 60 mg/kg body weight and injected intraperitoneal into rats during single STZ injection^24^. Blood glucose was monitored 48 h after STZ injection. To measure the inactive serum glucose level glucometer tapes (SD CODEFREE BLOOD GLUCOSE METER TEST STRIPS 50 T; SD Biosensor Inc, South Korea) were used on the rat tail vein. If the measured blood glucose was higher than 300 mg/dl, we considered diabetic rats^25^.

### Plant collection

Scrophularia striata (Teshnedari) is a regional plant which was collected from an altitude of 1427 m of the Zagros mountain chain (Ilam, Iran). A voucher specimen (No. 12-68353) is deposited in the Herbarium Laboratory of Islamic Azad University Science and Research Branch, Tehran, Iran and was identified by the same University. The floral part of the plant was used to attain the extraction, it is also worth mentioning that this plant grows and was collected in spring. We gained Permission for sample collection according to all the relevant institutional guidelines and legislation. The use of plants in the present study complies with international, national and/or institutional guidelines.

### Preparation of *Scrophularia striata* ethanolic extract

Dehydration is the primary step of all the steps of plant preparation for extraction. After collection, the plants needed to be cleaned with water even before the dehydration step. Then, the soaking method was used to extract the plant material. The soaking method was performed twice to ensure efficient extraction of the material. Every 200 g of dried *Scrophularia striata* received 400 mL of ethyl alcohol (Merck, Germany), and their mixture was placed in darkness for 72 h. The mixture was passed through filter paper three times. After the last filtering stage, it was placed in a dark place to slowly evaporate the alcohol. The dried extract after preparation was stored at 0–4 °C in the laboratory refrigerator. Finally, the dried extract was dissolved in the propylene glycol (PROPYLENE GLYCOL FOOD GRADE; Pure Organic Ingredients, USA) to get the required doses (100, 200 and 400 mg/kg). each dose of 100, 200 and 400 mg/kg bw/day solved in 1CC of propylene glycol^23^.

### Experimental design

The rats were randomly divided into 11 groups (n = 6: healthy control group (receiving propylene glycol which is the solvent of the extracted medicine as solvent); diabetic control group (induced by 60 mg/kg body weight Streptozotocin); three experimental healthy groups (receiving the extract at 100, 200 and 400 mg/kg bw/day), three experimental treatment groups (receiving the extract at 100, 200 and 400 mg/kg bw/day), and three pretreatment groups (receiving the extract at 100, 200 and 400 mg/kg bw/day). In this experiment, the pretreatment groups were healthy at the beginning of the experiment. In the pretreatment groups, daily gavage was performed with solvent for 2 weeks before and after STZ injection at 3 specified doses. The other groups also underwent daily gavage with the extract solution and solvent for 4 weeks.48 h after STZ injection to the Diabetic control group, experimental treatment groups and the pre-treatment groups blood glucose level measured and diabetic rats were confirmed by measuring a blood glucose concentration of more than 300 mg/dL.

### Dissection and pancreas sample collection

After completing 4 weeks of medication for all the groups, the rats in each group were dissected with a heart under anesthesia, blood was taken directly from their hearts and then centrifuged. Blood samples were collected with a cardiac puncture, to measure some biochemical factors including fasting blood sugar (FBS), hemoglobin A1c (HbA1c), and insulin; the serum was separated quickly, and then were aliquoted and stored at − 70 °C. Additionally, the pancreatic tissues were fixed in 10% formalin for histopathological examination.

### Histological assessment of pancreas tissue

On the dissection day, the rat was fasted for 12 h to anaesthetize with ketamine (80 mg/kg) and xylazine (10 mg/kg)^26^. Cross-sections of all pancreas lobes were collected, fixed in coded jars containing formaldehyde buffer solution, and then embedded in paraffin. Sections 5 µm in size were stained with hematoxylin and eosin (H&E) and examined by light microscopy. The status of histological damage was considered for atrophy, fibrosis and regeneration status as negative, mild, moderate and severe^27^.

### Determination of serum FBS, HbA1c and insulin in the studied rats

After experiment period, all animals were weighed by a digital scale, then anaesthetized with ketamine (80 mg/kg) and xylazine (10 mg/kg) and blood samples were taken from their hearts. The serum was separated by centrifugation at 10,000×*g* for 10 min and hemoglobin A1c (HbA1c) was measured by latex enhanced immunoturbidimetric assay with an HbA1c quantitative detection kit (Pars Azmoon, Tehran, Iran) using a photometric method. Fasting blood sugar (FBS) was measured by a photometric method with a glucose quantity detection kit (GOD-PAP) (Pars Azmoon, Tehran, Iran), and serum insulin was measured by enzyme-linked immunosorbent assay using a mouse insulin ELISA kit (Mouse Insulin ELISA Kit (ab277390) according to the manufacturer’s instructions^28–30^.

### Assessment of PDX1 and Ins1 gene expression by quantitative real-time PCR

Total RNA was extracted from the samples immediately after sampling according to standard protocols using an RNA purification kit (GeneJET™ RNA Purification Kit # K0732, Thermo Scientific—Fermentas, Latvia). Total RNA was treated with DNase to remove contaminated genomic DNA using DNase deletion reagents (DNase I, RNase-free (# EN0521) Fermentas, Latvia), according to the manufacturers’ protocol. RNA quality was assessed by agarose gel electrophoresis and UV spectroscopy. The cDNA was synthesized using a first strand transcription cDNA synthesis kit (Thermo Scientific -Fermmentas, Latvia), according to the manufacturers’ protocol. After sequencing both *Pdx1* and *Ins1* gene types and finding specific sequences of the species, the primers were designed by oligo7 software and blasted on the NCBI website. The glyceraldehyde-3-phosphate dehydrogenase (GAPDH) gene was used for normalization as an endogenous reference gene. The primers were *Pdx1* forward 5′CCTTTCCCGAATGGAACCGA3′, *Pdx1* reverse 5′TTTTCCACGCGTGAGCTTTG3′ and *Ins1* forward 5′GTCAAACAGCACCTTTGTGGT3′ and *Ins1* reverse 5′AGAAACCACGTTCCCCACAC3′. Agarose gel electrophoresis was used to evaluate the predicted size of the PCR amplitudes of genes. Standard curves for each gene were prepared using serial dilutions (1:4) of cDNA fused from total RNA extracted from the samples. In each experiment, the value of R^2^ was the standard curve N0.99, and no-template control experiments resulted in the absence of a detectable signal. Quantitative RT-PCR was performed using SYBR Green (Thermo Scientific Maxima SYBR Green/ROX qPCR Master Mix (2×) # K0221, Thermo Scientific—Fermentas, Latvia). A triplicate method was performed for quantitative real-time PCR using a 7900HT Fast Real-Time PCR System with a Fast 96 weeks Block Module (Applied Biosystems, Foster City, CA, USA). PCR data were obtained by Sequence Detector Software (SDS version 2.3 Rev C Patch, Applied Biosystems) and quantified by the standard curve method. This software plotted the real-time fluorescence intensity and selected the threshold within the linear phase of the amplicon profile. The software plotted a standard curve of the cycle at threshold versus extracted RNA quantity. Samples were measured in one plate for one target gene and their C_t_ values were in the linear range of the standard curve. In the qPCR test, outliers or sample failures were repeated for each gene. The Pfafle formula is used to calculate the ratio. Quantitative real-time PCR and analysis of expression data were performed based on a previous study^31^.

### Chemicals, reagents and kits

Streptozotocin (STZ, S0130) was purchased from Sigma-Aldrich (USA). 0.1 M sodium citrate buffer (pH 4.5) was purchased from Biochemazone (Canada). Glucometer tape (SD CODEFREE BLOOD GLUCOSE METER TEST STRIPS 50 T) was purchased from SD Biosensor Inc (South Korea). Ethyl alcohol was purchased from Merck (Germany). Propylene glycol food grade was purchased from Pure Organic Ingredients (USA). Ketamine (Ketamine 10%) and Xylazine (Xylazine 2%) were purchased from Alfasan (The Netherlands). HbA1c quantitative detection kit and Glucose quantity detection kit (GOD-PAP) were procured from Pars Azmoon (Tehran, Iran). Mouse Insulin ELISA Kit (Mouse Insulin ELISA Kit (ab277390)) was purchased from Abcam (Cambridge, United Kingdom). RNA purification kit (GeneJET ™ RNA Purification Kit # K0732), DNase deletion reagents (DNase I, RNase-free (# EN0521)), Transcription cDNA synthesis kit and SYBR Green for Quantitative RT-PCR (Thermo Scientific Maxima SYBR Green / ROX qPCR Master Mix (2×) # K0221) were purchased from Thermo Scientific -Fermmentas (Latvia).

### Statistical analysis

All data were statistically analyzed by SPSS-20 software. After confirming the normality of the data, one-way analysis of variance (ANOVA-one way) and Tukey's test were performed. The results are presented as the mean ± S. E. M Statistical inference was used.

## Results

### Effects of *Scrophularia striata* ethanolic extract on FBS, HbA1c and insulin in serum

**Figure 1:**
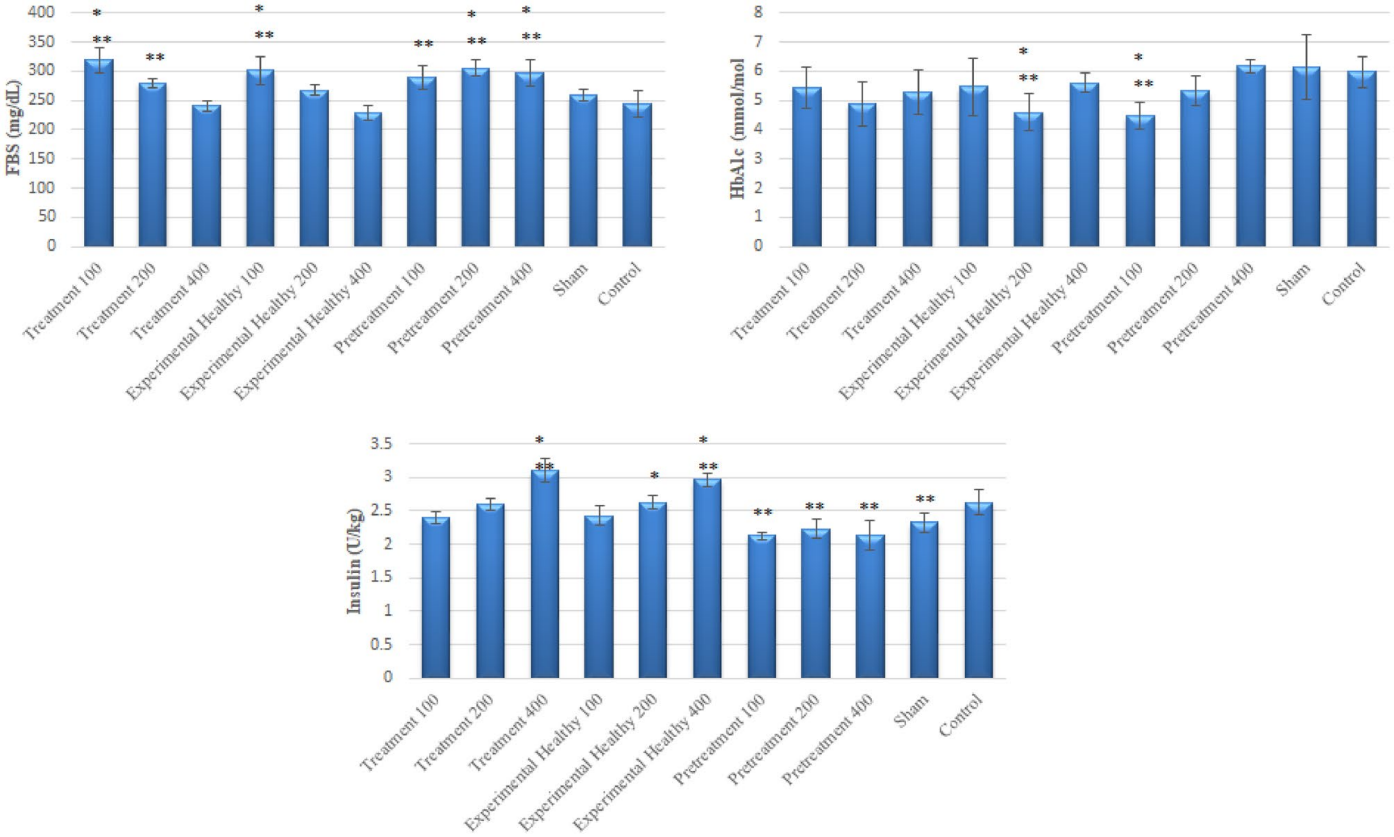
The effect of *S. striata* extracts on Insulin, FBS and HbA1c. Data are expressed as the mean ± SD; n = 6 for each group. 100, S. striata 100 mg/kg bw; 200*, S. striata* 200 mg/kg bw; 400, *S. striata* 400 mg/kg bw. Significant differences compared to the Sham and Control groups were shown *P* ≤ 0.05 *, and *P* ≤ 0.05**, respectively. ANOVA and Tukey’s were applied to evaluate the data.

According to Fig. 1, FBS and HbA1c increased in the sham group compared to the control group. In addition, FBS was significantly reduced in the 400-treatment group compared to the sham and control groups (*P* = 0.71 and *P* = 1, respectively). On the other hand, HbA1c index reduced in all the treatment, experimental healthy and pretreatment groups compared to the sham and control groups, which was significant in 200 healthy experimental and 100 pretreatment experimental groups (*P* = 0.01 and *P* = 0.005). However, insulin decreased significantly in the sham group compared to the control group (*P* = 0.018), and all the treatment and experimental healthy groups increased the insulin index significantly in a dose-dependent manner.

### Effects of *Scrophularia striata* ethanolic extract on *Pdx1* and *Ins1* gene expression

**Graph 1:**
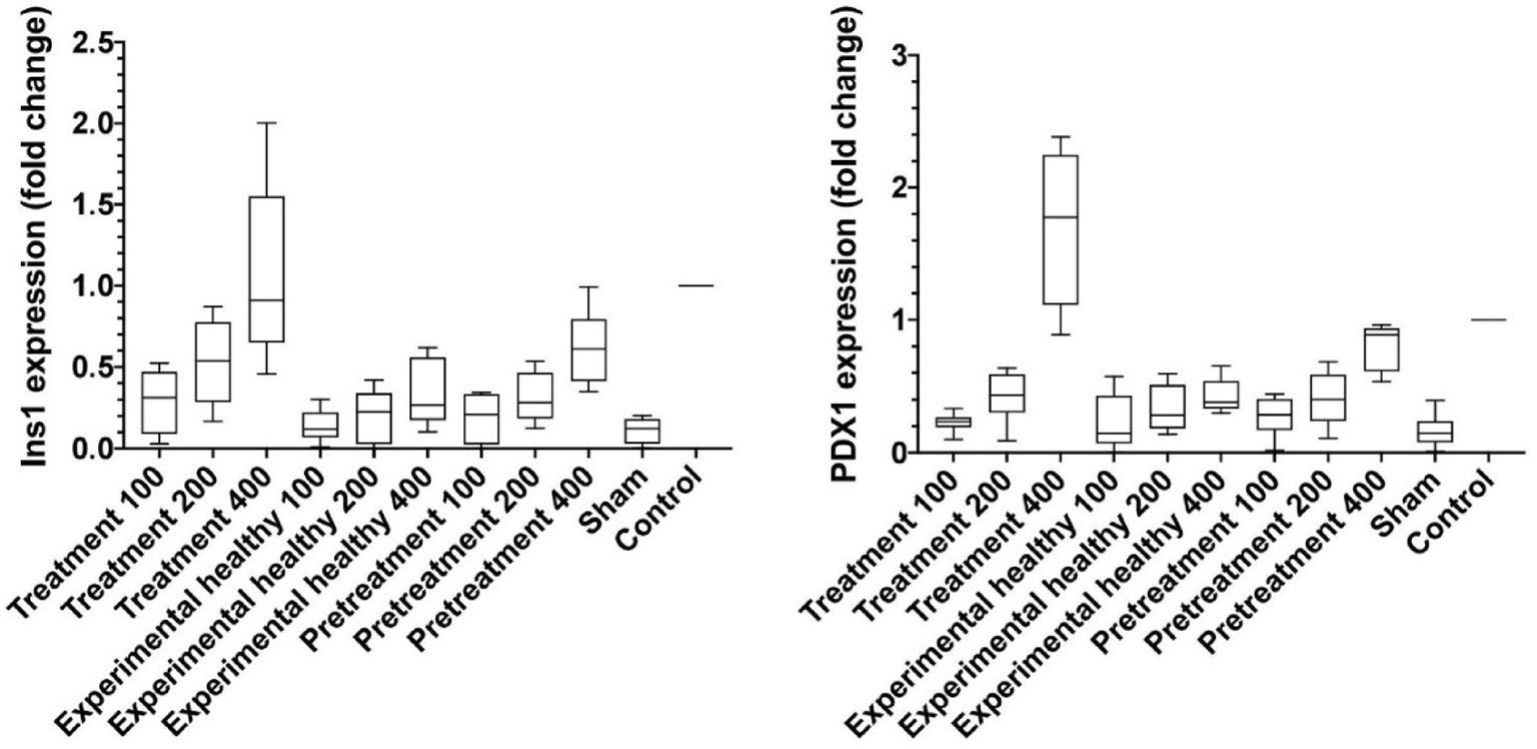
The results of gene expression.

Gene expression was compared between groups based on ∆∆ ct and fold change. According to an analysis of ∆∆ ct, when the ∆∆ ct value in a group is low gene expression will be higher. In addition to fold change, whatever fold change value in a group is high, therefore gene expression will also be high. As a result, in the sham group, *Pdx1* and *Ins1* gene expression-compared to that in the control group. In all of the other groups, *Ins1* gene expression was increased compared to that in the sham group. Otherwise, *Pdx1* gene expression just increased in the treatment 400 group compared to the sham group. The effect of *S. striata* was dose-dependent (Tables 1, 2, 3 and Graph 1).

**Table 1:** PDX1 and Ins1 gene expression in the studied rats.

Group	Gene expression fold change, median (IQR)	
	PDX1	Ins1
Treatment 100	0.234 (0.192–0.268)	0.313 (0.091–0.470)
Treatment 200	0.435 (0.303–0.590)	0.535 (0.286–0.774)
Treatment 400	1.775 (1.113–2.245)	0.911 (0.650–1.550)
Experimental healthy 100	0.144 (0.071–0.431)	0.119 (0.069–0.223)
Experimental healthy 200	0.283 (0.184–0.511)	0.225 (0.027–0.341)
Experimental healthy 400	0.381 (0.333–0.538)	0.267 (0.175–0.558)
Pretreatment 100	0.287 (0.171–0.405)	0.211 (0.024–0.335)
Pretreatment 200	0.401 (0.239–0.588)	0.283 (0.185–0.465)
Pretreatment 400	0.887 (0.611–0.936)	0.610 (0.412–0.794)
Sham	0.146 (0.077–0.238)	0.122 (0.030–0.181)
Control	1.00 (1.00–1.00)	1.00 (1.00–1.00)

**Table 2:** Pairwise comparison of PDX1 expression between groups based on Mann–Whitney.

*P* value	Treatment 100	Treatment 200	Treatment 400	Experimental healthy 100	Experimental healthy 200	Experimental healthy 400	Pretreatment 100	Pretreatment 200	Pretreatment 400	Sham	Control
Treatment 100	–	0.055	0.004	0.522	0.522	0.006	0.522	0.055	0.004	0.150	0.002
Treatment 200	0.055	–	0.004	0.200	0.337	0.810	0.150	1.000	0.010	0.078	0.002
Treatment 400	0.004	0.004	–	0.004	0.004	0.004	0.004	0.004	0.010	0.004	0.040
Experimental healthy 100	0.522	0.200	0.004	–	0.200	0.109	0.423	0.109	0.006	0.873	0.002
Experimental healthy 200	0.522	0.337	0.004	0.200	–	0.200	0.873	0.522	0.006	0.055	0.002
Experimental healthy 400	0.006	0.810	0.004	0.109	0.200	–	0.200	0.873	0.010	0.025	0.002
Pretreatment 100	0.522	0.150	0.004	0.423	0.873	0.200	–	0.200	0.004	0.150	0.002
Pretreatment 200	0.055	1.000	0.004	0.109	0.522	0.873	0.200	–	0.016	0.037	0.002
Pretreatment 400	0.004	0.010	0.010	0.006	0.006	0.010	0.004	0.016	–	0.004	0.002
Sham	0.150	0.078	0.004	0.873	0.055	0.025	0.150	0.037	0.004	–	0.002
Control	0.002	0.002	0.040	0.002	0.002	0.002	0.002	0.002	0.002	0.002	–

**Table 3:** Pairwise comparison of Ins1 expression between groups based on Mann–Whitney Test.

*P* value	Treatment 100	Treatment 200	Treatment 400	Experimental healthy 100	Experimental healthy 200	Experimental healthy 400	Pretreatment 100	Pretreatment 200	Pretreatment 400	Sham	Control
Treatment 100	–	0.109	0.006	0.150	0.423	0.631	0.337	0.749	0.037	0.109	0.002
Treatment 200	0.109	–	0.055	0.010	0.037	0.200	0.055	0.150	0.631	0.010	0.002
Treatment 400	0.006	0.055	–	0.004	0.004	0.010	0.004	0.006	0.109	0.004	0.305
Experimental healthy 100	0.150	0.010	0.004	–	0.522	0.078	0.522	0.037	0.004	0.749	0.002
Experimental healthy 200	0.423	0.037	0.004	0.522	–	0.337	0.749	0.262	0.006	0.337	0.002
Experimental healthy 400	0.631	0.200	0.010	0.078	0.337	–	0.262	0.873	0.055	0.037	0.002
Pretreatment 100	0.337	0.055	0.004	0.522	0.749	0.262	–	0.262	0.004	0.337	0.002
Pretreatment 200	0.749	0.150	0.006	0.037	0.262	0.873	0.262	–	0.025	0.016	0.002
Pretreatment 400	0.037	0.631	0.109	0.004	0.006	0.055	0.004	0.025	–	0.004	0.002
Sham	0.109	0.010	0.004	0.749	0.337	0.037	0.337	0.016	0.004	–	0.002
Control	0.002	0.002	0.305	0.002	0.002	0.002	0.002	0.002	0.002	0.002	–

The gene expression was calculated based on fold change by.

ΔCt = Ct (target gene) − Ct (Housekeeping gene).

ΔΔCt = ΔCt (Experimental sample) − ΔCt (Normal sample).

Gene expression = 2 − ΔΔ.

### Effects of *S. striata* ethanolic extract on histopathology of the pancreas

Pancreas histopathologic results are shown in Table 4. Histological study showed that the pancreas tissues of the control group had negative atrophy and fibrosis status and regeneration status. The pancreas tissues in the sham group showed moderate in atrophy and fibrosis status and negative in regeneration status.

**Table 4:** Histopathological results in the studied rats.

Group	Atrophy and fibrosis status	Regeneration status
Treatment 100	Negative	Negative
Treatment 200	Negative	Negative
Treatment 400	Negative	Negative
Experimental healthy 100	Negative	Suspicious positive
Experimental healthy 200	Negative	Suspicious positive
Experimental healthy 400	Negative	Suspicious positive
Pretreatment 100	Negative	Negative
Pretreatment 200	Mild	Negative
Pretreatment 400	Negative	Negative
Sham	Moderate	Negative
Control	Negative	Negative

**Figure 2:**
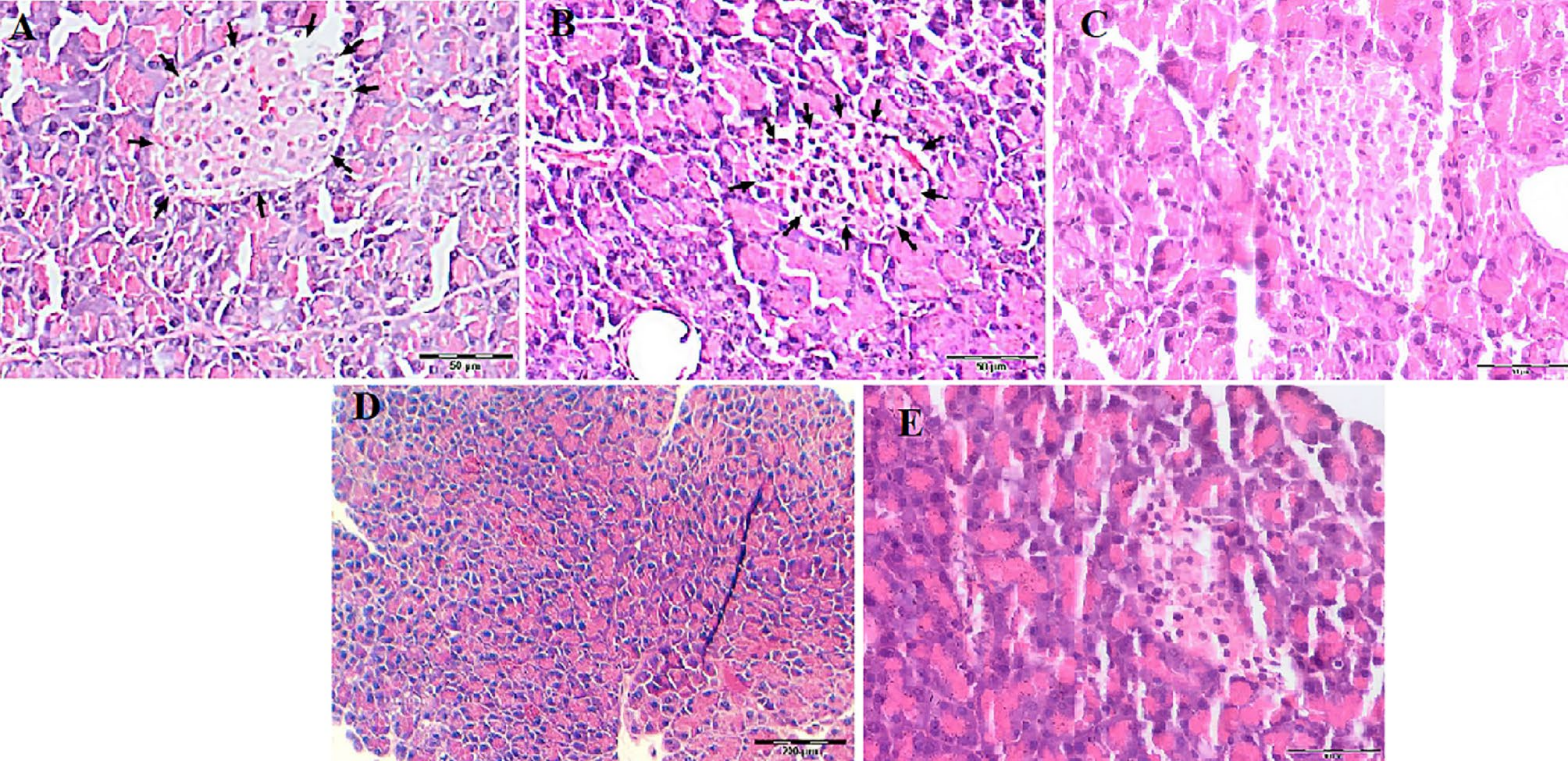
Histopathological findings of the effects of *Scrophularia striata* ethanolic extract on pancreas tissues of studied groups. 28 days after the experiment, pancreas tissues samples were evaluated Harris-Eosin hematoxylin staining (**A**) Sham, (**B**) Control, (**C**) Experimental healthy (healthy + *Scrophularia striata* 400 mg/kg), (**D**) Pre-treatment (treated before and after STZ injection with *S. striata* 400 mg/kg), (**E**) Treatment (diabetic + *S. striata* 400 mg/kg). No histopathological changes were observed in pancreas tissues with sham (**A**) and experimental healthy (**C**) islets of Langerhans (arrows) and ascites is seen in a normal pattern. In the control group (**B**) degeneration in the cells of the islets of Langerhans (arrows), and atrophy and fibrosis in the structure of the pancreas is shown. In the pre-treatment group (**D**) normal condition of the ascites is shown. All these lesions were markedly attenuated in *S. striata* treatment (400 mg/kg) (**E**) (H&E *200).

As shown in Fig. 2, no significant tissue changes were observed in sham group (Fig. 2A), which were healthy and experimental healthy group treated with 400 mg/kg *S. striata* extract (Fig. 2C); whereas the diabetic control group was observed degeneration in Langerhans islet cells and some degree of atrophy and fibrosis was seen in the structure of the pancreas (Fig. 2B). In the pretreatment group (400 mg/kg *S. striata* extract) was observed normal the condition of the ascites (Fig. 2D). Finally, in the treatment group (400 mg/kg *S. striata* extract) improved the islets of Langerhans in cellularity compared to the control group and are somewhat structurally closer to the sham group, which indicates the effectiveness of the treatment (Fig. 2E).

## Discussion

Persistent and chronic hyperglycemia reduces antioxidant defense system activity and therefore causes the production of free radicals. Increased levels of free radicals along with the failure of the natural antioxidant system generally cause cellular dysfunction and death. Oxidative stress is generated in diabetic situations and may be involved in the development of beta-cell dysfunction in the pancreas^32^.

Despite numerous studies on the pathogenesis of diabetes mellitus (DM) and new therapeutic strategies in recent years, the molecular mechanisms of DM pathogenesis and the antidiabetic action of drugs remain largely unknown. In the present investigation, STZ diabetic rats fed *Scrophularia striata* ethanolic extract (at doses of 100, 200 and 400 mg/kg bw) per day for 4 consecutive weeks exhibited significant blood glucose and hemoglobin Ac1 (HbA1c) reductions and increases in blood insulin levels. These observations are in agreement with a study that showed increased blood glucose concentrations in STZ-hyperglycemic rats compared to control rats^33^. This hypoglycemic potential, in addition to confirming the traditional use of this plant as a blood glucose-lowering agent has been reported in the literature^34^. Phytochemical studies on *Scrophularia striata* have shown the presence of compounds such as flavonoids, cinamic acid, phenylpropanoid, neptrin, flavonoid glycoside, acteoside1, quercetin and phenylpropanoid glycoside^35–37^. However, to date, the biological activity of these compounds has not been reported to have a hypoglycemic effect. Flavonoids are a group of phenolics of secondary plants with strong antioxidant properties. Independent studies have shown that flavonoids have antidiabetic effects^38^. Therefore, the same group of compounds (e.g., flavonoids) is predicted to respond to the antidiabetic and antioxidant properties of *S. striata*. Our results indicated that *Scrophularia striata* ethanolic extract can produce a dose-dependent stimulation of insulin release in rats.

The effect of hypoglycemia in plants may be because of the presence of insulin-like substances in plants, the stimulation of B cells to produce more insulin, the fiber found in plants at high levels to interfere with the absorption of carbohydrates or the effect of plants regenerative on pancreatic tissue^39–41^. Theoretically, hypoglycemic plants work through various mechanisms, such as modifying insulin sensitivity, increasing glucose-dependent insulin secretion, and stimulating islet regeneration of Langerhans in the pancreas of STZ-induced diabetic rats. In addition, the role of antioxidant compounds in the protection and treatment of diabetes has been remarked upon in various scientific studies. For instance, treating diabetic animals injected with STZ with N-acetyl-L-cysteine (NAC), a known antioxidant, prohibits high blood sugar by reducing oxidative stress and restoring beta-cell function^38^. On the other hand, STZ has been shown to cause selective damage to pancreatic beta-cells^42^. Hence, beta cell insulin content decreased in untreated diabetic control rats; however, in the *Scrophularia striata* treatment groups, this pancreatic insulin content was augmented.

There is ample evidence that β-cell regeneration and function in the adult pancreas are mediated partly by pancreatic and duodenal homobox 1 (*Pdx1*), and the changes in its expression are associated with alterations in the expression of target genes, including insulin 1 (*Ins-1*)^4^. In two previous studies, *Pdx1* and a subset of other major islet-enriched transcription factors were found to be significantly decreased in human diabetes mellitus type 2 (T2DM) islet β-cells^43,44^. In this study, we also indicated that the *Scrophularia striata* ethanolic extract increased *Ins-1* gene expression. The increased expression levels of the *Pdx1* and *Ins1* genes may be related to the methylation effect of *Scrophularia striata* in streptozotocin-induced diabetic rats. However, further investigations are needed to evaluate the methylated genes involved in the regeneration of the pancreas by *Scrophularia striata* administration.

## Conclusion

According to the results of the present study, the antidiabetic effects of the *S. striata* ethanolic extract were confirmed. In addition, it observed that the *S. striata* extract has the effect of reduction in FBS and HbA1c, in addition it has an increasing effect on serum insulin. Moreover, it was observed that the studied extract was effective in the pancreas by increasing *Pdx1* and *Ins1* gene expression. Generally, *S. striata* ethanolic extract, because of its antioxidant properties, can be considered a beneficial medicine in treatment of diabetes and pancreatic tissue complications due to diabetes. Further study of the detailed mechanisms is needed. This study has confirmed the other studies about the antidiabetic and protective properties of *S. striata* in diabetes. Therefore, additional studies can confirm these findings.
